# Evaluation of Retinal Changes Using Optical Coherence Tomography in a Pediatric Case of Susac Syndrome

**DOI:** 10.4274/tjo.27482

**Published:** 2017-01-17

**Authors:** Mehmet Kola, Hidayet Erdöl, Sevil Ertuğrul Atasoy, Adem Türk

**Affiliations:** 1 Karadeniz Technical University Faculty of Medicine, Department of Ophthalmology, Trabzon, Turkey

**Keywords:** optical coherence tomography, Retina, retinal artery occlusion, Susac syndrome, diagnosis

## Abstract

Susac syndrome is a rare occlusive vasculopathy affecting the retina, inner ear and brain. The cause is unknown, although it generally affects young women. This syndrome can be difficult to diagnose because its signs can only be revealed by detailed examination. These signs are not always concomitant, but may appear at different times. This report describes a pediatric case who was diagnosed with Susac syndrome when retinal lesions were identified in the inactive period with the help of optical coherence tomography (OCT). The purpose of this case report is to emphasize the importance of OCT in clarifying undefined retinal changes in Susac syndrome.

## INTRODUCTION

Susac syndrome (SS) is a relatively rare disorder characterized by the triad of encephalopathy, sensorineural hearing loss, and branch retinal artery occlusion (BRAO).^1^ It was first described in 1979 by Susac, and Hoyt further refined its description in 1986.^2,3^ The condition usually affects females in the mid-teen age group, though it can reportedly develop in individuals between 7 and 70 years old. To date, approximately 300 cases have been reported worldwide, but the prevalence of the disease is not exactly known.^1^ BRAO, one of the ocular signs of SS, is a common pathology; it generally develops bilaterally and affects multiple retinal fields. In the active stage, BRAO is best assessed by fundus fluorescein angiography (FFA), in which it typically appears as multifocal fluorescence in the arteriole walls.^4^ However, in the inactive stage, FFA is not particularly useful in diagnosing earlier retinal pathologies.^2^ This case report is presented to highlight the importance of optical coherence tomography (OCT) in the evaluation of inactive retinal changes in the inactive stage of SS.

## CASE REPORT

**Figure 1:**
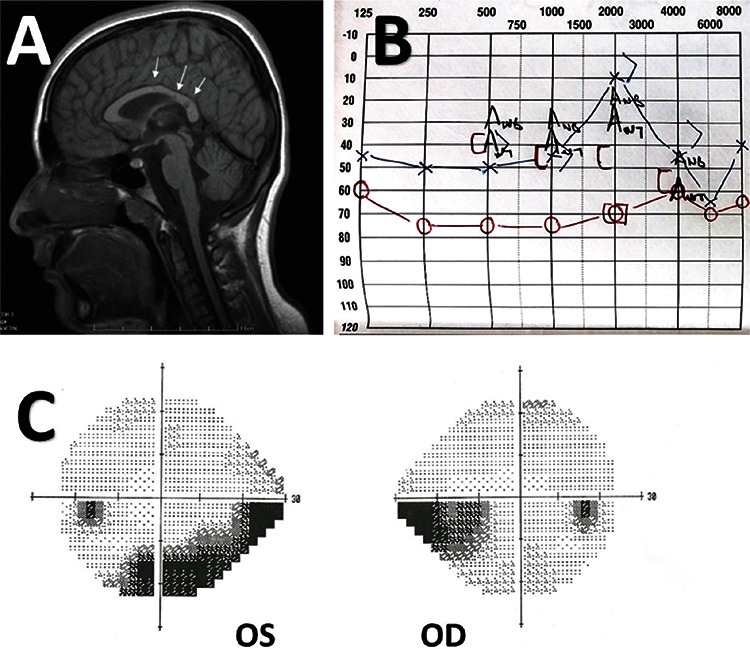
A pediatric Susac syndrome patient. A) T1-weighted magnetic resonance imaging shows hypointense corpus callosum lesions (arrows); B) odiometric analysis reveals bilateral sensorineural hypoacusis; C) visual field analysis shows bilateral scotoma secondary to previous branch retinal artery occlusion OS: Oculus sinister, OD: Oculus dexter

A 14-year-old female patient with a 2-year history of headaches and subsequent hearing loss was referred to our clinic for further testing and treatment for visual symptoms that had worsened over recent months. The patient’s history included visual complaints accompanied by clumsiness and difficulty walking which started about 3 months prior to her presentation to our clinic. Cranial magnetic resonance imaging (MRI) revealed lesions in the corpus callosum consistent with chronic infarct. Furthermore, odiometric analysis showed bilateral sensorineural hypoacusis (Figure 1).

**Figure 2:**
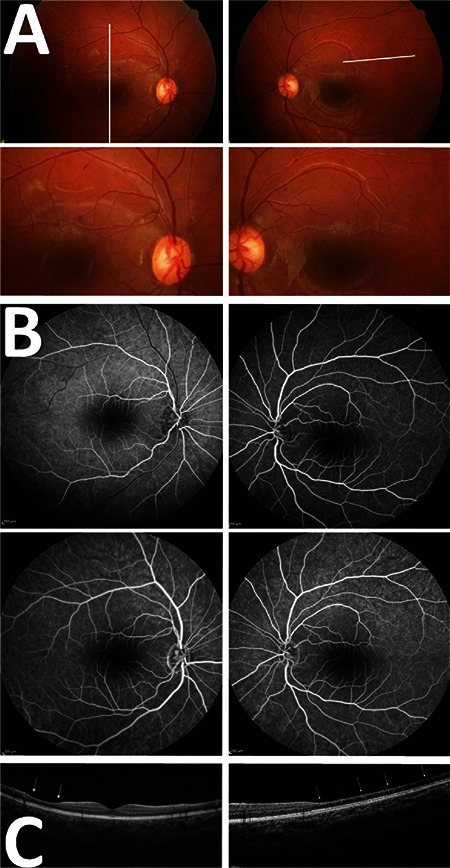
A pediatric Susac syndrome patient. A) Fundus photography; B) fundus fluorescein angiography images are normal in early and late phases; C) Optical coherence tomography sections taken from the areas marked with white lines on the fundus photographs. The arrows indicate atrophy of the inner retinal layers which emerged late secondary to branch retinal artery occlusion

Visual acuity was 20/20 in both eyes and intraocular pressure was 16 mmHg in the right eye and 17 mmHg in the left eye. Anterior and posterior biomicroscopic examination findings were normal. No clear pathology was apparent on FFA examination, but a partial defect was noted on visual field analysis (Figure 1). In both eyes, cross-sectional OCT revealed pronounced atrophic changes in the inner retinal layers corresponding to the areas of visual field loss (Figure 2). These findings were considered sequelae to previous BRAO. Taken together with her clinical signs, the patient was diagnosed with SS. The patient was followed without any treatment for about 1 year, during which no new active findings were observed.

## DISCUSSION

The etiology of SS is still not fully understood. It is believed to be an immunologic endotheliopathy that affects the microvasculature of the brain, retina, and inner ear. However, other unsupported theories such as vasospastic phenomena, coagulation disorders, and viral infections have also been implicated in its development.^2,5,6^ The disease tends to affect precapillary arterioles, and encephalopathy is usually the first clinical sign. The other clinical signs may emerge at various times after the development of encephalopathy. In about 10% of cases, disease onset occurs during pregnancy.^1,7^

Various clinical examinations and analyses are useful in the diagnosis of SS. Cranial MRI of SS patients performed due to neurological symptoms reveals infarct in the corpus callosum.^8,9^ Areas of infarct in the corpus callosum were also observed in the present case on MRI.

Odiologic tests which reflect inner ear involvement are also informative in SS patients.^6^ In our case, odiologic test results indicated neurosensorial hypoacusis in both ears. Another common finding in SS is partial visual field defects. This sign occurs as a result of BRAO, which is often present in the syndrome. In addition to visual field loss, ophthalmoscopy in these patients may reveal signs of retinal vasculitis, BRAO and optic atrophy.^2,6,10^ In the retinal vasculitis type, the refractive or nonrefractive yellowish Gass plaques which may be evident in the retinal arterioles are an important diagnostic finding for the disease. These plaques may sometimes be mistaken for embolism. On FFA examination, hyperfluorescent changes may also be observed in the retinal arterial walls in sections distant from areas of BRAO.^4,11,12,13^

BRAO seen in SS is generally bilateral and affects multiple retinal fields. In the active stage, BRAO is best recognized by FFA. In the chronic stage, however, the chance of overlooking retinal pathologies secondary to BRAO is fairly high, even in ophthalmologic examinations that include FFA.^2,4^

OCT enables retinal imaging comparable to histologic sections, and is currently used in the evaluation of many ophthalmologic conditions.^14,15,16^ In retinal artery occlusion, OCT examination shows increased retinal layer thickness and reflectivity in the short term, and is used to follow atrophy in these retinal layers in the long term.^14,17,18^ We also found in the current study that OCT examination provided useful information regarding retinal atrophic changes secondary to BRAO in SS. Brandt et al.^19^ also utilized OCT to evaluate atrophic changes in the retina arising in SS. They reported that the morphologic changes revealed by retinal OCT examination may facilitate the differential diagnosis of SS and multiple sclerosis.^19^

There is currently no definitive treatment protocol for SS. General treatment approaches based on the autoimmune causes of SS heavily feature immunosuppressive and immunomodulatory agents in the active stage.^1,20^ As the findings in the present case were considered chronic stage sequellae of SS, no treatment was administered.

## CONCLUSION

The diagnosis of SS can be difficult because its clinical signs do not always manifest concurrently. Therefore, a detailed history and thorough systemic evaluation are mandatory. Cranial MRI, odiologic tests and retinal imaging are important in the diagnosis of this syndrome. Especially after an attack, retinal changes secondary to BRAO that are not evident in ophthalmoscopic examination or FFA can be diagnosed by a detailed OCT examination based on visual field analysis.

### Ethics

Peer-review: Externally peer-reviewed.
